# Phonological similarity in the serial recall task hinders item recall, not just order

**DOI:** 10.1111/bjop.12575

**Published:** 2022-06-12

**Authors:** Steven Roodenrys, Dominic Guitard, Leonie M. Miller, Jean Saint‐Aubin, Jeffrey M. Barron

**Affiliations:** ^1^ School of Psychology University of Wollongong Wollongong New South Wales Australia; ^2^ École de psychologie Université de Moncton Moncton New Brunswick Canada

**Keywords:** phonological similarity, serial recall, short‐term memory

## Abstract

The phonological similarity effect in short‐term memory (STM) is the finding that serial recall of lists of similar sounding items is poorer than that of dissimilar sounding items. This is traditionally argued to be due to a detrimental effect on memory for the order of the words in the list and not on memory for the words themselves. Models that propose forgetting from STM is due to interference must invoke an additional compensatory process where the shared element of the words acts as a cue to recall, in order to account for the lack of an effect on memory for the words. However, the possibility of a detrimental effect of phonological similarity on item memory when these compensatory processes are not available has not been investigated. Two experiments (*n* = 60 and *n* = 57) in which similarity is operationalized in a way that precludes usage of compensatory processes are reported. The results clearly demonstrate that item recall is poorer for similar lists than dissimilar lists when similarity is defined in this way.

## BACKGROUND

The phonological similarity effect, poorer serial recall of lists of words that sound similar to each other than of lists of dissimilar words, is a cornerstone of the literature on short‐term memory (STM) that has shaped theories of memory for over half a century. First described by Conrad and Hull (1964) by comparing the recall of acoustically similar and dissimilar letters, it was quickly determined that the effect of similarity was to disturb the order of recall in the serial recall task, rather than the likelihood of retrieving the items at some point during recall (Wickelgren, 1965). This claim that similarity impairs the recall of order information but not item information has persisted through the literature ever since (e.g., Baddeley et al., 2018; Poirier & Saint‐Aubin, 1996; Watkins et al., 1974) and was recently included in a list of benchmark findings for short‐term and working memory and has major theoretical ramifications (Oberauer et al., 2018). In the present study, for the first time, we provide clear, direct evidence against that claim by systematically manipulating phonological similarity in two experiments in a manner that is free of potential confounds, after carefully reviewing the most relevant literature.

Short‐term memory is an area where there are numerous computational models that attempt to simulate human memory performance, and models can be pitted against each other to determine which provides the closest approximation to human data (e.g., Oberauer & Lewandowsky, 2008). Generally, these models are constructed such that phonological similarity disturbs the order of recall but does not reduce the likelihood of recalling an item, simulating human data when recalling lists of letters (e.g., Burgess & Hitch, 1999). However, this is potentially an over‐simplification with important theoretical consequences as these models are potentially wrong if similarity does have a negative impact on item information. The purported lack of an effect on item recall is, in fact, contradicted by some findings in the literature and may reflect the manner in which phonological similarity has been operationalized, and the methodology used, in previous research.

## PAST PHONOLOGICAL SIMILARITY DEMONSTRATIONS

We begin with a careful examination of previous studies on phonological similarity, focussing on the manner in which similarity has been operationalized, and the impact on recall of the identity of the items and the order of the items in the list. The focus is on studies that have used consonant‐vowel‐consonant (CVC) words as stimuli in serial recall, which are summarized in Table 1, as the experiments reported below use such stimuli and the review demonstrates a number of key issues. Although there are a number of ways in which it is possible to define phonological similarity, the most common operational definitions have probably been to use lists of rhyming stimuli (usually letters), or a manipulation where all the CVC words share a common vowel, and each consonant in a word is shared with at least one other word in the list that Gupta et al. (2005) described as canonical, because of its' widespread use. What all the definitions have in common is that all the items share at least one phoneme with all the other items in the list.

**TABLE 1:** Pattern of similarity effects of studies using CVC word stimuli with different definitions of similarity

Similarity	Potential retrieval cue for similar lists	Repetition of cue across lists	Difference on item recall	Difference on serial recall	Studies
Rhyming	Rime unit (VC)	All lists used the same set of words	Dissimilar = similar	Dissimilar > similar	Fallon et al. (1999) Exp. 2^a^
			Dissimilar > similar	Dissimilar > similar	Gupta et al. (2005) Exp 3 & 4^a^
		Not repeated	Dissimilar < similar	Dissimilar > similar	Nimmo and Roodenrys (2004) Exp. 1
			Dissimilar < similar	Dissimilar = similar	Gupta et al. (2005) Exp. 1 & 2 Fallon et al. (1999) Exp. 1
Canonical	Vowel	All lists used the same set of words	Dissimilar > similar	Dissimilar > similar	Coltheart (1993) Exp. 1^a^ Fallon et al. (1999) Exp 2^a^ Gupta et al. (2005) Exp 3 & 4^a^
		Not repeated	Dissimilar > similar	Dissimilar > similar	Coltheart (1993) Exp. 1 Fallon et al. (1999) Exp. 1 Gupta et al. (2005) Exp 1 & 2 Nimmo and Roodenrys (2004) Exp. 3
			Dissimilar = similar	Dissimilar > similar	Watkins et al. (1974)
	Initial C	Not repeated	Dissimilar = similar	Dissimilar > similar	Nimmo and Roodenrys (2004) Exp. 1
	Final C	Not repeated	Dissimilar = similar	Dissimilar > similar	Nimmo and Roodenrys (2004) Exp. 2
Alliterative	Onset (CV)	All lists used the same set of words	Dissimilar > similar	Dissimilar > similar	Gupta et al. (2005) Exp 3 & 4^a^
		Not repeated	Dissimilar = similar	Dissimilar > similar	Gupta et al. (2005) Exp. 1 & 2 Nimmo and Roodenrys (2004) Exp. 2
Consonants	Consonant frame (C_C)	Not repeated	Dissimilar = similar	Dissimilar > similar	Nimmo and Roodenrys (2004) Exp. 3

^a^ This study used the set of eight dissimilar words from Baddeley (1966). Only four of these words are CVC. Two are CV and one is CCV. One (bar) is CV or CVC depending on dialect.

### Rhyming

One very common operational definition of phonological similarity has been to make all the stimuli in the list rhyme. Before describing the research on CVC words, it is worth noting that the view that phonological similarity only affects the order of recall may have arisen because many of the early studies used letters as stimuli, and this practice is still common. Letters afford the selection of a familiar set of stimuli that rhyme, and a set that are relatively dissimilar, but this means they are necessarily drawn from a small set with repeated presentation of stimuli across lists. This methodology is likely to reduce item errors in recall and maximize order errors.

Focussing on those studies that have used CVC word stimuli, Table 1 presents a summary, grouping the studies on a number of characteristics, including which manipulation of similarity they used, and which component was the same across all the words in a list, offering a potential retrieval cue for similar lists (e.g., knowing that all the words rhyme). The third characteristic is whether this potential cue was the same for each list because the items are always drawn from a small pool, or whether it differed for each list because items came from an open set with no repetition across lists.

As can be seen from Table 1, the studies using a rhyme manipulation have not been entirely consistent in their results. Fallon et al. (1999) Experiment 2, using a stimulus set of eight words, found poorer recall of the rhyming lists than dissimilar lists when scored in position, but equivalent recall when scored without respect to position, supporting the claim that similarity affects memory for order but not identity. Gupta et al. (2005), also with a small set of stimuli, found poorer recall of rhyming than dissimilar lists when scored with respect to position and also when scored without respect to position (Experiments 3 & 4). These two papers used the same set of dissimilar words first used by Baddeley (1966), but different rhyming sets of eight words.

When the rhyme was unique to each list and the results were scored by a strict in‐position criterion, Nimmo and Roodenrys (2004, Experiment 1) found the typical advantage for the dissimilar lists; however, Fallon et al. (1999, Experiment 1) and Gupta et al. (2005, Experiments 1 and 2) both found recall of the rhyming lists to be just as good as the dissimilar lists. In contrast, the effect in scoring for item information only was consistent, with all three papers reporting an advantage for the rhyming lists.

An intuitively appealing explanation for this pattern is that the rhyme can be used as a cue to help recall the words in the list (Fallon et al., 1999; Nairne & Neumann, 1993). As the set of words in a rhyming list will often include most of the words that share that rhyme, it provides a very good cue to the identity of the words in the list, resulting in better recall of item information compared with the dissimilar condition. However, if the same words are used repeatedly across lists, this cue loses its value as the participant learns the sets of words that are being used in the different conditions.

### Canonical

The manipulation of phonological similarity described as canonical (Gupta et al., 2005) was introduced by Baddeley (1966), and his stimuli have been used in many papers since. His stimulus set consisted of eight CVC words with all the words sharing a vowel and a limited set of consonants, with two initial consonants and four final consonants across the eight words (*mad*, *man*, *map*, *mat*, *cad*, *can*, *cap* and *cat*). Unfortunately, his data were reported only for the number of entire sequences recalled correctly, so do not provide evidence regarding the impact on recall of items without respect to position. However, Coltheart (1993) used Baddeley's (1966) sets of eight stimuli and did find significantly poorer recall of items in the similar condition than in the dissimilar condition (77% vs. 96%, Experiment 1), even when the items were presented repeatedly across lists, offering the opportunity for the participants to learn the set. Fallon et al. (1999, Experiment 2) and Gupta et al. (2005, Experiments 3 & 4) report the same comparison with the same result. However, it should be noted that Baddeley's (1966) dissimilar set of eight words were not all CVC words, which may make them easier to recall (see the note to Table 1).

Coltheart (1993) also used a larger set of stimuli, all with the same vowel, but with no repetition of words across the lists and still found a significant decrement in recall of the items (77% vs. 85%, Experiment 1). Although supporting the view that phonological similarity should hinder item recall, Coltheart's results could be due to a confounding factor – interlist similarity. In effect, all of her similar lists used the same vowel (*a*) which may have created a level of confusion between items across similar lists, which was not present for the dissimilar lists. Consistent with this alternative explanation, Coltheart's results also revealed that half of the difference in recall of the similar and dissimilar items could be accounted for by explicit intrusions of items from the previous lists. It is worth noting that Fallon et al. (1999, Experiment 1) and Gupta et al. (2005, Experiments 1 & 2) used Coltheart's (1993) stimuli and also reported poorer item recall in the similar lists when items were not repeated, but neither reported an analysis of the errors.

In contrast, Watkins et al. (1974) found equivalent item level recall for similar and dissimilar lists. In the similar condition, all the words in a list shared a vowel, but a different vowel on each list. Although all the words were single syllable, some lists contained words that were not CVC in structure, which may reduce their similarity to CVC words, and only five different consonant sounds (b, k, d, p and t) were used in the set of 140 stimuli. As the same items were used in the similar and dissimilar condition, it is arguable that the similarity of the consonants was the same across the two conditions. Since the vowel in each similar list is consistent, this redundancy may provide a cue that counters any interference on the vowel, eliminating any differences in difficulty between conditions.

Nimmo and Roodenrys (2004) had the same manipulation of similarity, in that all the words in the list shared one phoneme and a small number of phonemes was used in the other positions, but the position of the shared phoneme differed across experiments. In their Experiment 3, where all words shared the vowel, they found a detrimental effect on item recall, and unlike the papers above, it was a different vowel on each list, ruling out the possibility that it was due to interference from previous lists. In their Experiment 1, a similar list had a common initial consonant across all the CVC words in the list, and each vowel and final consonant was shared with some other list words. In Experiment 2, all words had the same final consonant and shared the initial consonant and vowel with some other words in the list. In both experiments, item recall did not differ from the dissimilar condition. This clearly shows that the effect on recall depends on which component of the words is common to all words in the list.

### Alliterative

Gupta et al. (2005) used this term to refer to a definition of similarity where each word in a list of CVC words shared the initial consonant and the vowel. In terms of the proportion of the word that is common to all words in the list (two out of three phonemes), this is the same as in a rhyme definition and only the position of the shared phonemes differs, but the effect on recall is not the same. When they used the same small set of stimuli across trials (Experiments 3 & 4), they found that recall in correct position was poorer for the alliterative condition than the dissimilar condition and that this was also the case for item‐only scoring. This is inconsistent with the claim that similarity only affects order and not item information; however, it must be noted again that the dissimilar set were not all CVC words, which may provide an additional advantage in recall. However, when all items were new on each trial, the pattern was consistent with this claim. Both Gupta et al. (2005, Experiments 1 & 2) and Nimmo and Roodenrys (2004, Experiment 2) reported poorer recall in correct position for alliterative lists, but equivalent recall of items. This suggests that the alliterative component (CV) is not as effective a cue to recall the identity of the items as the rhyme.

### Consonants

There is one paper in the literature, which operationalized similarity in terms of all three possible combinations of two phonemes from the CVC, across different experiments. In their Experiment 3, Nimmo and Roodenrys (2004) included a similar condition in which all the CVC words shared the two consonants and had a unique vowel. The results showed the typical impairment on recall when scored in position, and no significant difference when scored for item recall, consistent with the claim that similarity affects order but not item memory.

### Summary of the review

To summarize, although the claim that phonological similarity does not impact item information has been prevalent in the literature, support for this view is rather thin. This is important because of the central role the phonological similarity effect has played in theorizing about STM (Baddeley, 1986; Oberauer et al., 2018). The picture has been somewhat complicated by the use of different manipulations of phonological similarity, but even when the same definition has been used, it is not consistently reported that item recall is equivalent to the dissimilar condition. The results when a small set of items are sampled repeatedly across the lists are actually relatively consistent in showing poorer item recall as well as ordered recall. However, this is not as convincing as it might seem because so many of them have relied on the same set of words where the dissimilar set are not uniformly CVC, which may make them easier to recall than CVC words, or may reflect something idiosyncratic about those particular sets of words.

When items are new on each trial, there are studies which show equivalent recall when scored in correct position and several that show impaired recall. However, when scoring for item recall, all three possible outcomes in comparing similar and dissimilar word lists have been found across different operational definitions of similarity. An explanation for these different outcomes in the literature described above may lie in how effectively different components of the word (e.g., CV vs. VC vs. V) function as a cue. It is even possible that a cue may impair recall in comparison with a dissimilar list if the cue is actually inefficient and its use taxes the system. It is often stated that words are more readily cued by their beginning than other components. Nelson and Garland (1969) demonstrated that learning of visual paired associate CVC words was quicker if the stimulus and response words shared the initial consonant than the vowel, and argued this is because it provides more information. If a participant tries to make use of an uninformative cue, such as a common vowel across the items, it may hinder recall relative to the dissimilar list. The studies reviewed suggest the rhyme is the most effective cue. All of the studies described above have used a similar condition that involves some component that is common across all words in the list, thus offering the opportunity to use that component as a cue in recall. It should be noted that cueing by a common component of the list words is a useful aid to item recall but insufficient to benefit order recall. By this account, the common component cues all words in the list equally and is not linked to specific positions. However, it seems likely that it would have a greater effect on the recall of items in later positions where recall is poorer.

In addition, many studies have used dissimilar conditions that allowed for some words in a list to share phonemes, which might be expected to reduce the difference in item recall if interference does occur. What appears to be critical in assessing the claim that similarity does not affect item recall in STM is observing a detrimental effect in the absence of other possible explanatory factors. From the review above, only Experiment 3 of Nimmo and Roodenrys (2004) appears to do so, despite having the vowel as a potential cue to recall. The purpose of the following experiments is to examine the impact on serial recall performance when phonological similarity is operationalized without list‐wide redundancy and the potential for compensatory factors such as the use of an effective cue for all the list items.

### The present study

The experiments reported below aim to provide a test of the assertion that phonological similarity influences order memory but not item memory. More specifically, the two experiments involve four conditions, the second experiment being a replication of the first with a second set of stimuli. The first condition is the control, dissimilar one. In the first experiment, some lists have some phonemes occurring twice in a list in this condition but in different intrasyllabic positions, whereas in the second experiment no phoneme was repeated within a six‐item list (e.g., *tap*, *hug*, *gym*, *boss*, *cord* and *rail*). Nimmo and Roodenrys (2004) utilized a dissimilar condition like this, but it is not clear that many other studies have gone to such lengths, and some have clearly allowed phonemes to occur more than once in a dissimilar list (e.g., Baddeley et al., 2018). Allowing repeated phonemes in the dissimilar condition would reduce the difference on item recall if interference does operate within the lists, so previous studies may have been biased against finding an effect. The second condition is the typical rhyme condition where each word in the list rhymes with the others (e.g., *net*, *jet*, *vet*, *pet*, *bet* and *debt*). In the other two conditions, each word shares two of its three phonemes with another word in the list. Each possible pair is shared in two of the six words in a list. The two conditions differ in how they are shared. In the *similar consistent* condition, the two phonemes of a word are shared with another word, so two words in the list rhyme, two share the two consonants, and two share the initial consonant and vowel (e.g., *bone*, *leaf*, *pad*, *reef*, *patch* and *barn*). In the *similar inconsistent* condition, the two phonemes of each word are shared with two different words in the list so no word shares more than a single phoneme with any other word (e.g., *cap*, *wipe*, *mob*, *rub*, *cot* and *rice*). Therefore, the potential for a phoneme to be lost from a word, all other things being equal, is the same across the two similar conditions, but the impact is expected to be different. In the *consistent* condition, the loss of one of the three phonemes from a word will make it confusable with one other word in the list. For instance, in the example provided above, the loss of the first phoneme */l/* of the word *leaf,* but not the loss of the phoneme /*i/* or /*f*/ will make it indistinguishable from *reef*. However, this is not the case for the *inconsistent* condition where any two phonemes of the word are sufficient to discriminate it from the other words in the list. For instance, in the example above, after losing any phoneme of the word *cap*, it will still be possible to distinguish it from all other list items.

We seek to examine the effect of phonological similarity between words in the lists when all the words in the list share some phonemes but no phoneme is shared across all the words. The primary aim is to determine whether phonological similarity between items that does not provide a reliable cue has a detrimental effect on recall of the words, irrespective of position. It is predicted that recall in correct serial position will be worse in the rhyme condition than the dissimilar condition, but item recall will be better in the rhyme condition than the dissimilar condition, as found in previous research (Fallon et al., 1999; Gupta et al., 2005; Nimmo & Roodenrys, 2004; Wickelgren, 1965). Critically, it is predicted that recall will be worse in the consistent and inconsistent similar conditions in comparison with the dissimilar condition.

## EXPERIMENT 1

### Method

#### Participants

Sixty‐one adult participants from the Prolific participant recruitment platform took part in the experiment. They were all native English speakers from North America, Britain or Australia with an approval rating of at least 90% on Prolific. They had a mean age of 33.7 years (*SD* = 10.1 years), and 35 were male. The study took approximately 10 min to complete, and participants were paid £1.50. The data from one participant were lost due to a problem with the computer system.

Based on the effect size (Cohen's *f* = 1.20) reported in Experiment 3 of Nimmo and Roodenrys (2004) who observed a lower item recall level for their similar items compared with their dissimilar items, we computed a sensitivity analysis to guide our sample size selection for both experiments. More exactly, a one‐way repeated measured sensitivity analysis was conducted with G*Power (Faul et al., 2009; Version: 3.1.9.4) with an alpha of 0.05 a power of 0.95 and the default parameters for the correlation between repeated measure and the non‐sphericity correction. The results from the analysis revealed that 60 participants would allow us to detect an effect size more than four times smaller than the original study on which our manipulation was based (Cohen's *f* = 0.24).

#### Stimuli

All the stimuli used were CVC words. Five lists of six words were created for each condition, as described above (see Appendix A). The words in each set were matched on several lexicosemantic dimensions (see Table A1) such as word frequency, concreteness, length and neighbourhood characteristics using Levenshtein distance (see Yarkoni et al., 2008, for a description). The phonological similarity of each word to all other words in its list was calculated using the metric of Mueller et al. (2003) which evaluates similarity in terms of articulatory features of the phonemes, and values closer to zero indicate greater similarity. A one‐way ANOVA was conducted to compare the within‐list similarity of the words in the different conditions. This found a significant effect, *F*(3, 116) = 172.58, *p* < .001, $\eta_{p}^{2}$ = 0.82. Tukey's HSD tests revealed the words in the rhyming lists were more similar to each other (*M* = 0.27, *SD* = 0.07) than the consistent and inconsistent conditions (*M* = 0.71, *SD* = 0.13, and *M* = 0.74, *SD* = 0.10, respectively), which did not differ from each other, but were more similar than the dissimilar condition (*M* = 0.87, *SD* = 0.13).

#### Procedure

A bespoke programme was used to run the experiment over the internet. On each trial, participants saw a fixation cross at the centre of the window and clicked a button on the screen to start the trial. They were then presented with six words in white on a black background, one after the other, in the centre of the screen at a rate of one word per second. Once the last word had been presented, it was replaced on the screen by a response box labelled ‘stimulus 1’ and participants typed their response before clicking another button to submit the response and move on to the next item. They were instructed that they could leave a response box blank when they could not recall the corresponding word. The first two trials were practice trials, one involving dissimilar words and one involving similar words, followed immediately by the 20 experimental trials. The trials were arranged in a different random order for each participant, and the words in each list were randomized for each participant.

### Results

**FIGURE 1:**
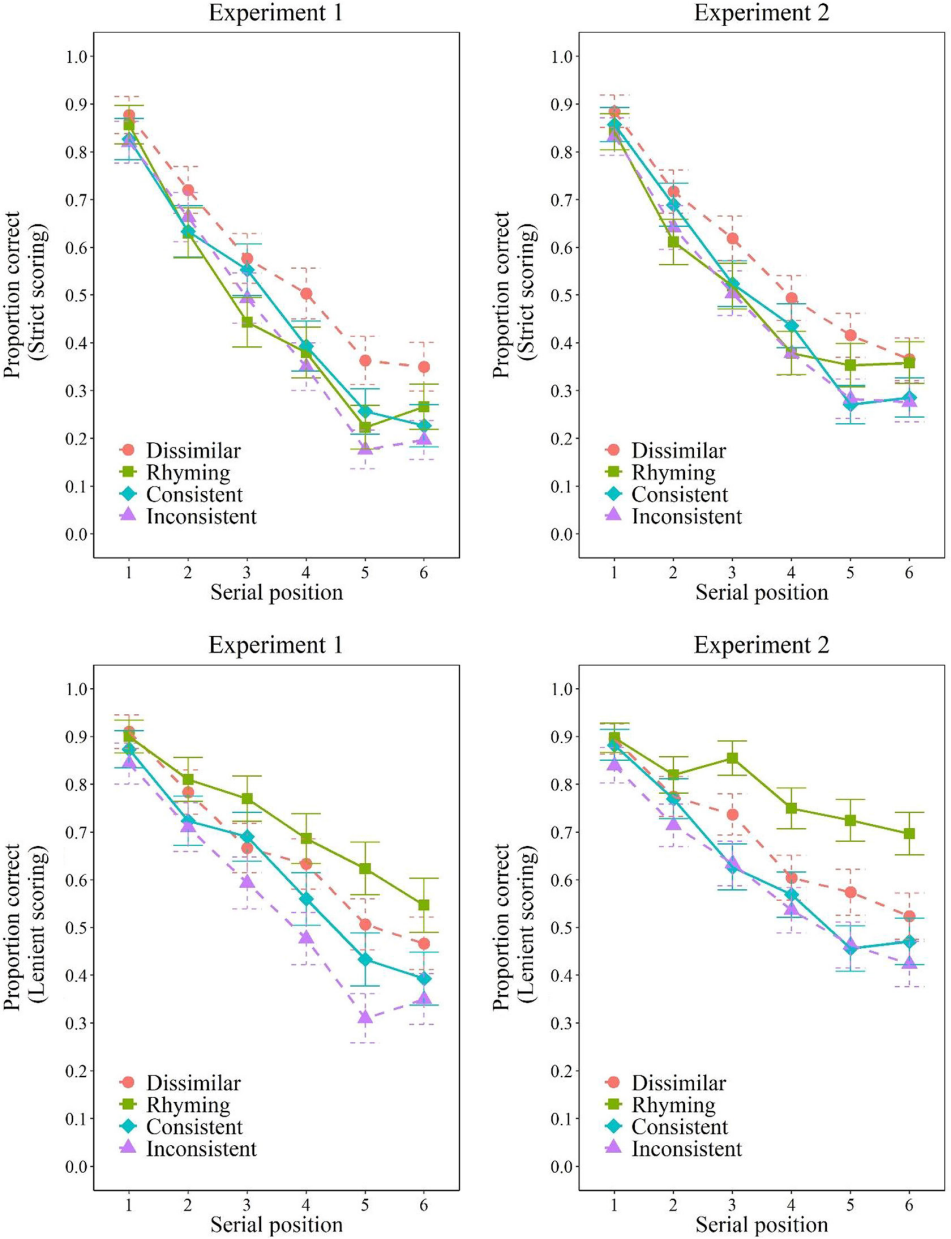
Proportion of correct responses (strict scoring: Top row; lenient scoring: Bottom row) for Experiment 1 (left column) and for Experiment 2 (right column). Error bars represent 95% within‐participant confidence intervals computed according to Morey's (2008) procedure

**FIGURE 2:**
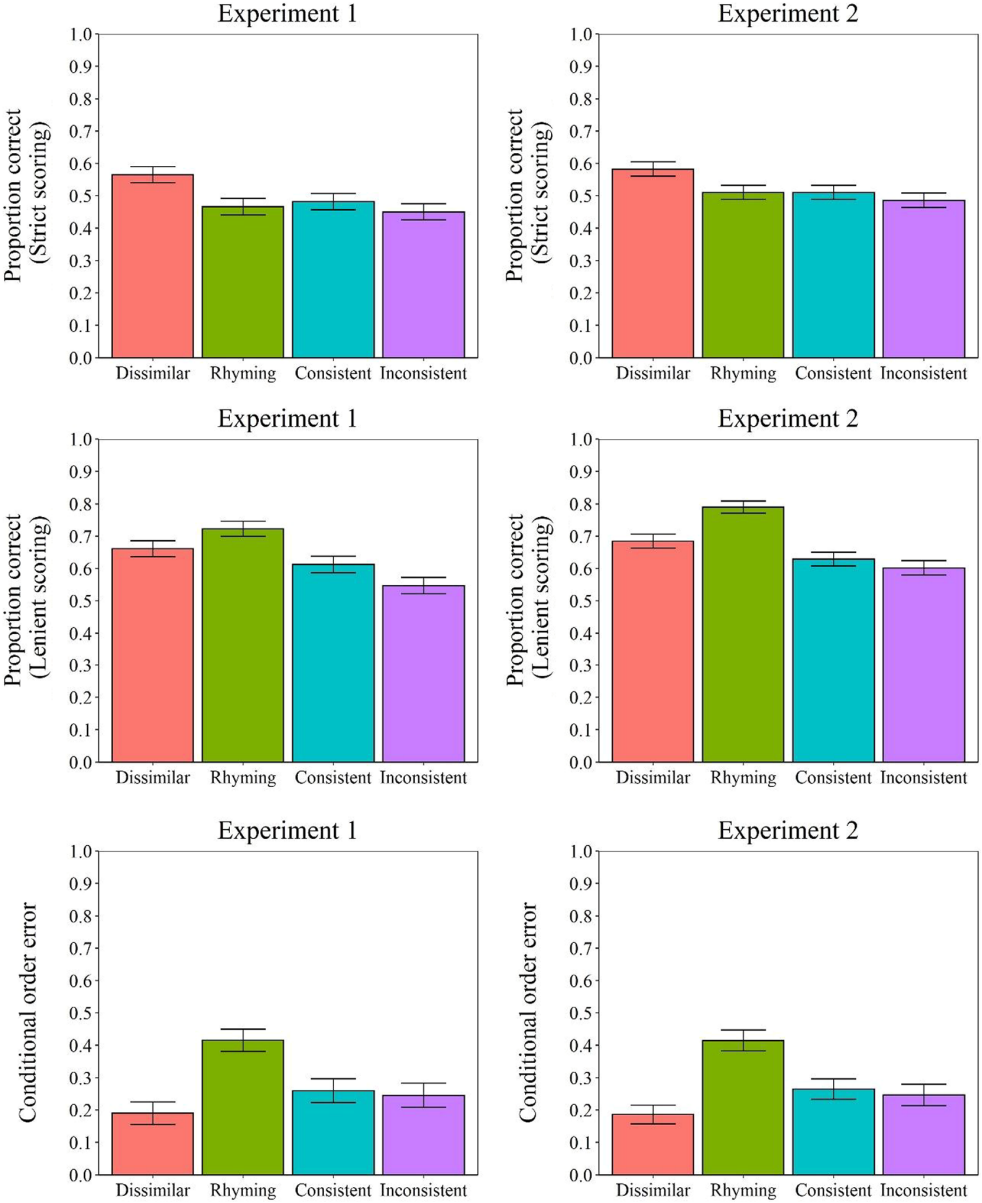
Proportion of responses (strict scoring: Top row; lenient scoring: Middle row; conditional error: Bottom row) for Experiment 1 (left column) and for Experiment 2 (right column). Error bars represent 95% within‐participant confidence intervals computed according to Morey's (2008) procedure

Data are available in the Open Science Framework repository, https://osf.io/u6nx5/?view_only=07aedb2a00854aaba76ab5c1412d1a43. Prior to analysis of responses, typographical and spelling errors were corrected. This included instances such as transpositions of letters within the word or pressing a key adjacent to the correct key resulting in a nonword response (e.g., ‘doke’ for ‘dole’). Homophones of the presented word or misspellings that preserved the phonology were also scored as correct (e.g., wail and whale). In total, 1.3% of items were corrected. Over half of these were in the rhyming condition where the rhyming words appear to have primed the incorrect spelling.^1^ In both the experiments, participants' responses were scored via a strict scoring criterion and a lenient scoring criterion. According to a strict scoring criterion, the to‐be‐remembered items had to be recalled in their presented position to be considered correct. According to a lenient scoring criterion, the to‐be‐remembered items had to be recalled in any position to be considered correct. Proportion of conditional order errors were also computed for both experiments by dividing the number of order errors, when a word presented is recalled out of position, by the number of words presented that were recalled regardless of their order (Poirier & Saint‐Aubin, 1996). The proportion of responses was then assessed as a function of serial position (1 to 6) and condition (dissimilar, rhyming, consistent and inconsistent) via repeated measures analysis of variance (ANOVA). Results for Experiment 1 and Experiment 2 are illustrated in Figures 1 and 2.

#### Strict scoring

Overall, recall performance of the participants when assessed via strict scoring was superior in the phonologically dissimilar condition (*M* = 0.56, *SD* = 0.29) compared with the other conditions. The performance of the participants was similar across the rhyming condition (*M* = 0.47, *SD* = 0.27), the consistent condition (*M* = 0.48, *SD* = 0.27) and the inconsistent condition (*M* = 0.45, *SD* = 0.28). The analysis of variance confirmed those trends. The analysis of variance revealed a main effect of condition, *F*(3, 177) = 11.91, *p* < .001, $\eta_{p}^{2}$ = 0.17, a main effect of serial position, *F*(5, 295) = 216.87, *p* < .001, $\eta_{p}^{2}$ = 0.79, and a two‐way interaction between those factors, *F*(15, 885) = 2.22, *p* = .005, $\eta_{p}^{2}$ = 0.04. Post‐hoc Tukey's HSD tests revealed the participants performed better in the dissimilar condition compared with all other conditions, all *p*s < 0.001, all Cohen's *d* > 0.529, which did not differ one from another, all *p*s > 0.423, all Cohen's *d* < 0.202. The interaction simply reflects the growing impact of phonological similarity as one moves towards the end of the list.

#### Lenient scoring

Participants' responses were assessed via lenient scoring, revealing that performance was superior when the to‐be‐remembered items were rhyming (*M* = 0.72, *SD* = 0.19) compared with the other conditions. Participants were also better in the dissimilar condition (*M* = 0.66, *SD* = 0.24) relative to the consistent condition (*M* = 0.61, *SD* = 0.21), which was superior to the inconsistent condition (*M* = 0.55, *SD* = 0.23). The analysis of variance confirmed these trends. Again, the analysis of variance revealed a main effect of condition, *F*(3, 177) = 33.65, *p* < .001, $\eta_{p}^{2}$ = 0.36, a main effect of serial position, *F*(5, 295) = 93.71, *p* < .001, $\eta_{p}^{2}$ = 0.61, and a two‐way interaction between those factors, *F*(15, 885) = 2.76, *p* < .001, $\eta_{p}^{2}$ = 0.05. Post‐hoc Tukey's HSD tests revealed the participants performed better in the rhyming condition compared with all other conditions, all *p*s < 0.003, all Cohen's *d* > 0.398. Post‐hoc analysis also revealed that participants performed better in the dissimilar condition compared with the consistent condition, *p* = .033, Cohen's *d* = 0.330 and that participants performed better in the consistent condition compared with the inconsistent condition, *p* = .002, Cohen's *d* = 0.439.

#### Conditional order errors

Examination of conditional order errors revealed that the proportion of order errors was lower in the dissimilar condition (*M* = 0.19, *SD* = 0.30) than the other conditions. This examination also revealed that the proportion of order errors was larger in the rhyming condition (*M* = 0.42, *SD* = 0.31) while the proportion of order errors was similar between the consistent condition (*M* = 0.26, *SD* = 0.32) and the inconsistent condition (*M* = 0.25, *SD* = 0.32). The analysis of variance confirmed the presence of a main effect of condition, *F*(3, 177) = 31.28, *p* < .001, $\eta_{p}^{2}$ = 0.35. Post‐hoc Tukey's HSD tests revealed that the proportion of order errors was larger in the rhyming condition compared with the other conditions, all *p*s < 0.001, all Cohen's *d* > 0.816. The proportion of order errors in the dissimilar condition was smaller than the proportion of order errors in the rhyming condition (*p* < .001, Cohen's *d* = 1.219), and in the consistent condition (*p* = .022, Cohen's *d* = 0.406), but not the inconsistent condition (*p* = .103, Cohen's *d* = 0.286). Then, post‐hoc analysis confirmed that the proportion of order errors was similar between the consistent condition and the inconsistent condition (*p* = .935, Cohen's *d* = 0.072).

#### Other errors

The proportion of responses made up of omissions, intrusions and repetitions of a list word during recall are shown in Table 2. An analysis of variance on the proportion of omissions confirmed a significant effect of condition, *F*(3, 177) = 9.20, *p* < .001, $\eta_{p}^{2}$ = 0.135. Post‐hoc Tukey's HSD tests were conducted on all comparisons but, for the sake of brevity, only significant differences will be reported. There were significantly more omissions in the consistent and inconsistent conditions than the dissimilar condition (*p* < .01, Cohen's *d* = 0.510, and *p* < .01, Cohen's *d* = 0.458, respectively), or the rhyming condition (*p* < .001, Cohen's *d* = 0.556, and *p* < .001, Cohen's *d* = 0.450, respectively).

**TABLE 2:** Mean proportion of errors (and standard deviations) by condition in experiments 1 and 2

Error type	Dissimilar	Rhyming	Consistent	Inconsistent
Experiment 1				
Omission	.15 (.16)	.15 (.13)	.21 (.17)	.21 (.19)
Intrusion	.17 (.14)	.08 (.07)	.16 (.12)	.22 (.14)
Repetition	.02 (.04)	.05 (.06)	.02 (.03)	.02 (.03)
Experiment 2				
Omission	.11 (.11)	.06 (.08)	.13 (.11)	.14 (.11)
Intrusion	.19 (.11)	.10 (.07)	.21 (.14)	.23 (.14)
Repetition	.02 (.02)	.05 (.03)	.03 (.03)	.02 (.03)

**TABLE A1:** Statistics for words used in Experiment 1

Statistic	FREQ	LgWF	LgCD	OLD	OLDF	PLD	PLDF	CNC	AoA	VM	AM	DM	NLET	NPHN	NSYL
Dissimilar words															
Mean	74.16	3.01	2.78	1.26	8.35	1.03	8.79	4.16	6.30	5.65	4.09	5.47	4.00	3.07	1.00
*SD*	108.04	0.84	0.73	0.18	0.25	0.10	0.49	1.01	2.43	1.14	0.82	0.87	0.00	0.25	0.00
Min	0.60	1.40	1.36	1.00	7.81	1.00	7.97	1.75	2.90	3.11	3.00	3.91	4.00	3.00	1.00
Max	467.02	4.25	3.75	1.65	8.77	1.50	9.98	5.00	12.24	7.51	6.59	6.89	4.00	4.00	1.00
*N*	30.00	30.00	30.00	30.00	30.00	30.00	30.00	26.00	30.00	26.00	26.00	26.00	30.00	30.00	30.00
Rhyming words															
Mean	70.91	2.84	2.60	1.32	8.33	1.02	8.91	3.83	6.53	5.26	3.95	5.16	4.00	3.10	1.00
*SD*	171.44	0.83	0.75	0.22	0.29	0.06	0.61	1.00	2.44	1.45	1.00	0.85	0.00	0.31	0.00
Min	0.71	1.18	1.11	1.00	7.89	1.00	7.81	1.85	3.32	2.33	2.24	3.45	4.00	3.00	1.00
Max	708.92	4.37	3.84	1.75	9.43	1.30	10.13	5.00	11.93	7.33	6.55	6.57	4.00	4.00	1.00
*N*	29.00	29.00	29.00	29.00	29.00	29.00	29.00	29.00	29.00	24.00	24.00	24.00	30.00	29.00	30.00
Consistent words															
Mean	62.32	2.93	2.70	1.23	8.36	1.04	8.89	4.16	6.69	5.07	3.93	5.34	4.00	3.00	1.00
*SD*	100.62	0.79	0.72	0.21	0.31	0.13	0.54	0.83	3.02	0.97	1.02	0.80	0.00	0.00	0.00
Min	0.77	1.34	1.30	1.00	7.66	1.00	7.96	1.73	2.94	3.40	1.67	2.89	4.00	3.00	1.00
Max	388.90	4.16	3.69	1.75	9.24	1.50	9.94	5.00	13.65	6.75	6.20	6.50	4.00	3.00	1.00
*N*	29.00	28.00	28.00	28.00	28.00	28.00	28.00	28.00	28.00	28.00	28.00	28.00	30.00	28.00	30.00
Inconsistent words															
Mean	45.28	2.90	2.67	1.27	8.36	1.06	8.98	4.10	6.61	5.00	3.73	5.23	4.00	3.03	1.00
*SD*	53.75	0.76	0.72	0.25	0.18	0.15	0.53	1.01	2.18	1.30	0.85	1.03	0.00	0.18	0.00
Min	1.37	1.57	1.30	1.00	8.03	1.00	7.51	1.53	3.44	2.24	2.62	2.77	4.00	3.00	1.00
Max	237.67	4.48	3.85	1.90	8.91	1.55	9.96	5.00	11.67	7.13	5.52	6.82	4.00	4.00	1.00
*N*	30.00	30.00	30.00	30.00	30.00	30.00	30.00	29.00	30.00	28.00	28.00	28.00	30.00	30.00	30.00
*F*	0.37	0.24	0.28	0.72	0.10	0.83	0.61	0.77	0.13	1.53	0.67	0.61	**–**	**–**	**–**
*p*	.773	.869	.838	.540	.960	.480	.612	.511	.945	.210	.575	.608	**–**	**–**	**–**

*Note*. FREQ = CELEX frequency (from Medler & Binder, 2005); LgWF = log frequency; LgCD = log contextual diversity (from Brysbaert & New, 2009); OLD = mean Levenshtein distance for the 20 closest orthographic neighbours; OLDF frequency of the 20 closest orthographic neighbours; PLD = mean Levenshtein distance for the 20 closest phonologic neighbours; PLDF frequency of the 20 closest phonologic neighbours (from Yarkoni et al., 2008); CNC = mean concreteness (from Brysbaert et al., 2014); AoA = age of acquisition (from Kuperman et al., 2012); VM = mean valence, AM = mean arousal, DM = mean dominance (from Warriner et al., 2013); NLET = number of letters; NPHN = number of phonemes; NSYL = number of syllables (from Coltheart, 1981). *N* indicates the number of words for which that measure was available, and f and p are the Anova value and probability of whether the four sets of words differ on that measure.

**TABLE B1:** Statistics for words used in Experiment 2

Statistic	FREQ	LgWF	LgCD	OLD	OLDF	PLD	PLDF	CNC	AoA	VM	AM	DM	NLET	NPHN	NSYL
Dissimilar words															
Mean	14.53	2.75	2.54	1.34	8.47	1.18	8.63	4.40	5.86	5.26	4.06	5.36	3.69	3.10	1.00
*SD*	14.15	0.39	0.35	0.30	0.44	0.25	0.69	0.69	1.75	1.26	0.90	0.87	0.75	0.30	0.00
Min	1.01	2.06	1.85	1.00	7.56	1.00	7.50	2.17	2.53	2.00	2.45	3.00	3.00	3.00	1.00
Max	77.70	3.80	3.41	2.00	9.71	1.90	10.84	5.00	11.50	8.23	6.27	7.11	6.00	4.00	1.00
*N*	42.00	42.00	42.00	42.00	42.00	42.00	42.00	42.00	42.00	40.00	40.00	40.00	42.00	42.00	42.00
Rhyming words															
Mean	16.35	2.75	2.54	1.30	8.61	1.11	8.89	4.37	6.55	5.19	3.88	5.23	3.67	3.14	1.00
*SD*	14.84	0.41	0.41	0.28	0.57	0.18	0.68	0.65	2.20	1.03	0.84	0.74	0.69	0.35	0.00
Min	1.79	1.86	1.76	1.00	6.94	1.00	7.85	2.72	3.47	1.95	2.62	3.35	3.00	3.00	1.00
Max	78.77	3.94	3.60	2.20	10.16	1.70	10.31	5.00	12.06	7.05	5.93	6.32	6.00	4.00	1.00
*N*	42.00	42.00	42.00	42.00	42.00	42.00	42.00	42.00	42.00	36.00	36.00	36.00	42.00	42.00	42.00
Consistent words															
Mean	14.09	2.73	2.50	1.30	8.54	1.11	8.79	4.43	6.61	5.11	3.67	5.31	3.67	3.10	1.00
*SD*	7.06	0.33	0.31	0.29	0.52	0.20	0.68	0.84	2.03	1.01	0.76	0.83	0.69	0.30	0.00
Min	2.68	2.10	1.90	1.00	7.31	1.00	7.45	1.93	3.17	2.62	2.58	3.30	3.00	3.00	1.00
Max	28.97	3.52	3.32	1.90	9.54	1.75	10.17	5.00	11.05	7.25	5.62	6.89	5.00	4.00	1.00
*N*	42.00	42.00	42.00	42.00	42.00	42.00	42.00	42.00	42.00	40.00	40.00	40.00	42.00	42.00	42.00
Inconsistent words															
Mean	16.83	2.77	2.58	1.27	8.42	1.08	8.81	4.34	6.59	5.33	3.80	5.39	3.79	3.10	1.00
*SD*	8.89	0.37	0.34	0.26	0.39	0.19	0.59	0.73	2.10	1.14	0.92	0.88	0.65	0.30	0.00
Min	5.41	2.00	1.88	1.00	7.74	1.00	7.70	1.61	3.15	2.47	2.33	3.14	3.00	3.00	1.00
Max	41.65	3.55	3.27	1.80	9.30	1.85	10.45	5.00	11.56	7.58	5.71	6.78	5.00	4.00	1.00
*N*	42.00	42.00	42.00	42.00	42.00	42.00	42.00	41.00	42.00	40.00	40.00	40.00	42.00	42.00	42.00
*F*	0.55	0.09	0.37	0.42	1.28	1.72	1.16	0.11	1.37	0.28	1.49	0.25	0.28	0.24	–
*p*	.648	.965	.776	.737	.283	.165	.327	.955	.254	.837	.220	.864	.839	.865	–

*Note*. FREQ = CELEX frequency (from Medler & Binder, 2005); LgWF = log frequency; LgCD = log contextual diversity (from Brysbaert & New, 2009); OLD = mean Levenshtein distance for the 20 closest orthographic neighbours; OLDF frequency of the 20 closest orthographic neighbours; PLD = mean Levenshtein distance for the 20 closest phonologic neighbours; PLDF frequency of the 20 closest phonologic neighbours (from Yarkoni et al., 2008); CNC = mean concreteness (from Brysbaert et al., 2014); AoA = age of acquisition (from Kuperman et al., 2012); VM = mean valence, AM = mean arousal, DM = mean dominance (from Warriner et al., 2013); NLET = number of letters; NPHN = number of phonemes; NSYL = number of syllables (from Coltheart, 1981). *N* indicates the number of words for which that measure was available, and f and p are the Anova value and probability of whether the four sets of words differ on that measure.

There was a significant effect of condition in the analysis of the intrusion proportions, *F*(3, 177) = 36.97, *p* < .001, $\eta_{p}^{2}$ = 0.385. Post‐hoc Tukey's HSD tests revealed that there were fewer intrusions in the rhyming condition than the other conditions (all *p* < .0001, and Cohen's *d* > 0.789). There were also significantly more intrusions in the inconsistent condition than the consistent or dissimilar conditions (*p* < .0001, Cohen's *d* = 0.538, and *p* < .001, Cohen's *d* = 0.571, respectively).

There was a significant effect of condition in the analysis of the repetition errors, *F*(3, 177) = 11.69, *p* < .001, $\eta_{p}^{2}$ = 0.165. Post‐hoc Tukey's HSD tests revealed that there were more repetitions in the rhyming condition than the other conditions (all *p* < .0001, and Cohen's *d* > 0.535).

### Discussion

The results of the first experiment clearly demonstrate the standard finding of better recall of items in their correct position when they were phonologically dissimilar to each other in comparison with any of the similar conditions. They also replicate the finding that recall of the items without respect to serial position is superior for rhyming lists over dissimilar lists (e.g., Gupta et al., 2005). Interestingly, despite the much lower level of similarity in the consistent and inconsistent condition compared with the rhyming condition, the impact on recall in the correct position did not differ as the overall level of recall was the same and lower than the dissimilar condition. This was despite the better recall of items in the rhyming condition and poorer recall of items in the consistent and inconsistent conditions, relative to the dissimilar condition.

The results of this experiment extend and help to clarify the previous findings in the literature by demonstrating that there is a detrimental effect of phonemic similarity on item recall when the similar condition does not include a component which is present in every word in the list, and very tight control is exercised over the stimuli. These results lend support to the previous findings in the literature of a deleterious effect of phonological similarity on item information (e.g., Coltheart, 1993; Fallon et al., 1999; Gupta et al., 2005; Nimmo & Roodenrys, 2004). A comprehensive discussion of the results will be provided after the second experiment.

## EXPERIMENT 2

The aim of the second experiment was to replicate the results of the first experiment with a different set of stimuli. This replication is needed to establish that the effects observed here do not depend on some peculiarities of the stimuli that will make the effects impossible to replicate in other laboratories or with other stimuli. This risk is well illustrated by the famous study by Baddeley et al. (1975) who equated short and long words on all dimensions except pronunciation time and found a better recall of short words over long words. The effect has been replicated many times with the original stimuli (e.g., Cowan et al., 1992), but all attempts with different stimuli developed with the same rules failed (e.g., Neath et al., 2003; Service, 1998). In addition, the new stimuli were developed to avoid repetition of any phonemes in the dissimilar condition. In order to achieve this aim and to generate more trials for each condition, we slightly relaxed the stringency of matching of the stimuli across conditions on the corpus‐based lexical variables (see Appendix B).

### Method

#### Participants

Fifty‐seven undergraduate Psychology students from an Australian University participated as part of a course requirement. The average age was 20.3 years (*SD* = 2.6 years), and 42 were female. As mentioned above, 60 participants were planned, but due to technical difficulties, 57 participants composed the final sample.

#### Stimuli

As in Experiment 1, all the stimuli used were CVC words. Seven lists of six words were created for each condition (see Appendix B). The sets of words were again matched on several lexicosemantic dimensions (see Table B1). A one‐way ANOVA was conducted to compare the within‐list similarity of the words in the different conditions, as in Experiment 1. This found a significant effect, *F*(3, 164) = 319.23, *p* < .001, $\eta_{p}^{2}$ = 0.85. Tukey's HSD tests revealed the words in the rhyming lists were more similar to each other (*M* = 0.27, *SD* = 0.06) than the consistent and inconsistent conditions (*M* = 0.74, *SD* = 0.11, and *M* = 0.74, *SD* = 0.10, respectively), which did not differ from each other, but were more similar than the dissimilar condition (*M* = 0.89, *SD* = 0.11).

#### Procedure

Except for the number of lists, the procedure of Experiment 2 was identical to Experiment 1.

### Results

The results of Experiment 2 are displayed in Figure 1 for strict and lenient scoring as function of condition and serial position, and in Figure 2 for the two former mentioned scoring approaches in addition to conditional order errors as a function of conditions. Responses were once again corrected for misspellings and obvious typographical errors. 1.7% of items were corrected.^1^

#### Strict scoring

When participants' responses were assessed via strict scoring, the performance of the participants was superior in the dissimilar condition (*M* = 0.58, *SD* = 0.28). Like Experiment 1, participants' performance was of comparable level across the rhyming condition (*M* = 0.51, *SD* = 0.29), the consistent condition (*M* = 0.51, *SD* = 0.28) and the inconsistent condition (*M* = 0.49, *SD* = 0.28). The analysis of variance revealed a main effect of condition, *F*(3, 168) = 9.30, *p* < .001, $\eta_{p}^{2}$ = 0.14, a main effect of serial position, *F*(5, 280) = 211.85, *p* < .001, $\eta_{p}^{2}$ = 0.79 and a two‐way interaction between those factors, *F*(15, 840) = 2.44, *p* = .002, $\eta_{p}^{2}$ = 0.04. Post‐hoc Tukey's HSD tests confirmed the descriptive trends. More exactly, the analysis revealed that participants' performance was superior in the dissimilar condition compared with all other conditions, all *p*s < 0.001, all Cohen's *d* > 0.426, which did not differ one from another, all *p*s > 0.575, all Cohen's < 0.209. Once again, the difference across conditions was larger for later than initial serial positions.

#### Lenient scoring

Overall, with the lenient scoring, the performance was superior in the rhyming condition (*M* = 0.79, *SD* = 0.17) compared with the dissimilar condition (*M* = 0.68, *SD* = 0.22), which was superior to both the consistent (*M* = 0.63, *SD* = 0.22) and the inconsistent conditions (*M* = 0.60, *SD* = 0.23). Once again, the analysis of variance confirmed those trends. Like Experiment 1, there was a main effect of condition, *F*(3, 168) = 69.54, *p* < .001, $\eta_{p}^{2}$ = 0.55, a main effect of serial position, *F*(5, 280) = 93.57, *p* < .001, $\eta_{p}^{2}$ = 0.63, and a two‐way interaction between those factors, *F*(15, 840) = 4.69, *p* < .001, $\eta_{p}^{2}$ = 0.08. Post‐hoc Tukey's HSD tests confirmed that all conditions differ one from another, all *p*s < 0.001, all Cohen's *d* > 0.503, except the inconsistent similar condition and the consistent similar condition which did not differ one from another, *p* = .212, Cohen's *d* = 0.312.

#### Conditional order errors

Exploration of conditional order errors revealed that the proportion of order errors in the dissimilar condition (*M* = 0.19, *SD* = 0.28) was again inferior to the other conditions. Echoing the results of Experiment 1, there were more order errors in the rhyming condition (*M* = 0.41, *SD* = 0.31) compared with the consistent condition (*M* = 0.26, *SD* = 0.31) and the inconsistent condition (*M* = 0.25, *SD* = 0.32). The analysis of variance confirmed the presence of a main effect of condition, *F*(3, 168) = 39.48, *p* < .001, $\eta_{p}^{2}$ = 0.41. Post‐hoc Tukey's HSD tests revealed that the proportion of order errors differ one from another in all four conditions, all *p*s < 0.038, all Cohen's *d* > 0.446, except the inconsistent condition and the consistent condition, *p* = .831, Cohen's *d* = 0.129.

#### Other errors

The proportion of responses made up of omissions, intrusions and repetitions of a list word during recall are shown in Table 2. An analysis of variance on the proportion of omissions confirmed a significant effect of condition, *F*(3, 168) = 21.876, *p* < .001, $\eta_{p}^{2}$ = 0.281. Post‐hoc Tukey's HSD tests were conducted on all comparisons but, for the sake of brevity, only significant differences will be reported. There were significantly fewer omissions in the rhyming condition than the other conditions (all *p* < .001, and Cohen's *d* > 0.568). There were also significantly fewer omissions in the dissimilar condition than the inconsistent condition (*p* < .05, Cohen's *d* = 0.380).

There was a significant effect of condition in the analysis of the intrusion proportions, *F*(3, 168) = 36.48, *p* < .001, $\eta_{p}^{2}$ = 0.394. Post‐hoc Tukey's HSD tests revealed that there were fewer intrusions in the rhyming condition than the other conditions (all *p* < .001, and Cohen's *d* > 0.914). There were also significantly more intrusions in the inconsistent condition than the dissimilar condition (*p* < .01, Cohen's *d* = 0.436).

There was a significant effect of condition in the analysis of the repetition errors, *F*(3, 177) = 17.11, *p* < .001, $\eta_{p}^{2}$ = 0.234. Post‐hoc Tukey's HSD tests revealed that there were more repetitions in the rhyming condition than the other conditions (all *p* < .0001, and Cohen's *d* > 0.448). There were also significantly more repetitions in the consistent condition than the dissimilar condition (*p* < .05, Cohen's *d* = 0.344).

### Discussion

The results of the second experiment are extremely similar to those of the first experiment, confirming the soundness of our findings. On the strict, in‐position scoring, performance was better in the dissimilar condition than the similar conditions, which did not differ from each other. On the lenient scoring, recall of the items was again significantly better if the list of words rhymed than if they were dissimilar and, again, significantly worse than the dissimilar condition in the other similarity conditions. The one difference of note is that in this experiment the level of item recall was equivalent between the similar consistent and similar inconsistent conditions.

## GENERAL DISCUSSION

The results of these two experiments clearly demonstrate that recall of the words in the list, independent of order, is impaired by phonological similarity when similarity is not defined by the rhyme. Some previous studies have also demonstrated this effect, but the current experiments extend on those by demonstrating that this is the case when there was no component common to all the items in a list that might act as a cue to recall the items, and free of proactive interference effects from having the same words presented in previous trials. In the similar conditions, two phonemes from each word were present in other words in the list, and in both experiments, these conditions were recalled significantly less well than the dissimilar condition. Contrary to the claim that similarity only affects the order of recall, the magnitude of the difference observed between these conditions in the lenient scoring is only slightly smaller than the difference observed in the strict scoring, suggesting much of the effect in the strictly scored data is due to the loss of information about the identity of the items, rather than an effect on order of recall.

The two similar conditions differ in how the phonemes were shared across the words in a list. In the consistent condition, the two phonemes of a word that were shared, were shared with one other word, so two words in the list rhymed, two shared the onset and vowel, and two shared the two consonants. In the inconsistent condition, the two phonemes of a word that were shared, were shared with different words so no two words overlapped on more than a single phoneme.

In Experiment 1, items in the consistent lists were better recalled than in the inconsistent lists (0.61 vs. 0.55), whereas in Experiment 2 they did not differ significantly (0.63 vs. 0.60), although the difference was in the same direction. In both experiments, the lower item recall in the inconsistent condition was mirrored by an increase in intrusions. It is possible that a small difference in recall, and intrusions, arises because on some lists the common component may assist in recall in a redintegration process after the degraded representations have been retrieved (Schweickert, 1993). For example, as the words in a list were randomized on each presentation, the two words which shared the onset, such as *pad* and *patch*, or the rhyme, might occur in sequence and if the participant notices, it may be an effective cue to recall the second word (cf. Nelson & Garland, 1969). This might provide a small boost to recall in the consistent condition that is statistically unreliable with the sample sizes used and may reflect differing rates of guessing across the experiments. Removing trials where related items occurred sequentially would leave too few trials to analyse reliably, so this notion needs to be tested with greater power by deliberately presenting lists in which the similar items are paired or separated.

Computational models of STM have modelled the effect of phonological similarity as impacting the order of recall, but not necessarily the probability of recalling an item anywhere in the list (e.g., Lewandowsky & Farrell, 2008). It remains to be seen whether they can model performance in the consistent and inconsistent similarity conditions in these experiments. One obvious difficulty is that the objectively less similar conditions show worse item recall than both the rhyming condition and the dissimilar condition. Additional assumptions will need to be made in order to capture the similarity manipulation in these experiments. Lewandowsky and Farrell (2008) compared how well three different models of serial recall could simulate their human data in recalling lists of letters. The representation of similarity in these simulations was based on auditory confusion matrices for all letters. The variation in results, shown in Table 1, when words share phonemes in different positions (e.g., when the vowel is common to all items vs. when a consonant is common) suggests that the similarity of words may not be captured simply by the confusability of individual phonemes across those words.

This paper is the first to have deliberately sought to challenge the widely accepted notion that phonological similarity affects only the order of recall and not the likelihood of recalling an item in serial recall. It demonstrates that items which share phonemes are less well recalled, irrespective of serial position, and therefore offers a new constraint on theories of STM.

## AUTHOR CONTRIBUTIONS

**Steven Roodenrys:** Conceptualization; formal analysis; investigation; methodology; project administration; writing – original draft; writing – review and editing. **Dominic Guitard:** Conceptualization; data curation; formal analysis; investigation; methodology; visualization; writing – original draft; writing – review and editing. **Leonie M. Miller:** Conceptualization; methodology; writing – original draft; writing – review and editing. **Jean Saint‐Aubin:** Conceptualization; methodology; writing – original draft; writing – review and editing. **Jeffrey M. Barron:** Investigation; software; writing – review and editing.

## CONFLICT OF INTEREST

All authors declare no conflict of interest.

## Data Availability

The stimuli are provided in the manuscript (see Appendices), and the data for all experiments are available on the Open Science Framework (https://osf.io/u6nx5/?view_only=07aedb2a00854aaba76ab5c1412d1a43).

## Footnotes

^1^
The data scored with spelling errors marked as incorrect were also analysed, and the same pattern of results was obtained.

## APPENDIX A

### A.1

The 120 words used in Experiment 1.

**Table:**

Dissimilar				
Ball	Bill	Main	Bead	Bull
lock	duck	port	wise	dome
pays	lace	wife	face	fort
wine	moon	sack	hock	hide
seem	pies	foes	mull	leak
ring	road	read	pert	cash

Rhyming				
born	sail	goal	hang	same
corn	mail	dole	rang	maim
dawn	fail	bowl	tang	came
horn	pail	foal	gang	lame
lawn	gail	soul	sang	dame
yawn	tail	pole	pang	game

Consistent				
mace	lime	pick	seal	dung
maze	line	pill	seat	dull
bide	hoot	mess	lurk	goat
bird	heat	mass	lick	gate
gout	wing	ride	case	rock
lout	ding	side	base	sock

Inconsistent				
coat	wake	lake	fate	fine
hole	bait	rate	rake	bile
fill	feet	beat	beak	rail
fawn	fern	bone	barn	reek
cage	wide	lice	foam	foot
gain	mine	vine	loan	hook

*Note*. The appendix presents which words were presented in each of the conditions (dissimilar, rhyming, consistent and inconsistent). Each row represents one list.

## APPENDIX B

### B.1

The 168 words used in Experiment 2.

**Table:**

Dissimilar						
Cheese	Gem	Loaf	Cart	Leak	Rail	Coin
rash	ban	cod	bin	goat	cord	doll
myth	sword	nap	pearl	ditch	hug	jam
bell	pile	gum	maze	rob	tap	kit
jug	cave	torch	rag	pain	gym	wave
hop	wig	web	fed	hush	boss	rug

Rhyming						
hail	thorn	vat	vet	shin	sip	dung
gale	horn	rat	jet	pin	chip	hung
whale	warn	bat	pet	gin	dip	lung
veil	lawn	mat	bet	thin	hip	rung
bail	torn	chat	debt	chin	lip	sung
nail	corn	pat	net	tin	tip	tongue

Consistent						
bull	wit	gut	reef	womb	fog	hen
wool	pit	hut	leaf	tomb	log	den
fake	hull	nod	pad	cage	porch	tool
fame	hum	knock	patch	cane	pork	tooth
pan	raid	soap	bone	peg	beard	ram
pen	rod	soup	barn	pig	bid	rim

Inconsistent						
moss	tag	lock	seed	cot	sack	dome
shade	dam	bomb	lean	mob	ham	folk
lace	comb	palm	lap	cap	hid	fan
sheep	turf	lid	tape	rub	shark	rum
rot	beef	port	code	rice	sheet	shine
rip	keen	ward	cake	wipe	weed	ripe

*Note*. The appendix presents which words were presented in each of the conditions (dissimilar, rhyming, consistent and inconsistent). Each row represents one list.
